# A novel integrase-containing element may interact with Laem-Singh virus (LSNV) to cause slow growth in giant tiger shrimp

**DOI:** 10.1186/1746-6148-7-18

**Published:** 2011-05-14

**Authors:** Wattana Panphut, Saengchan Senapin, Siriporn Sriurairatana, Boonsirm Withyachumnarnkul, Timothy W Flegel

**Affiliations:** 1 Centex Shrimp, Faculty of Science, Mahidol University, Rama 6 Road, Bangkok 10400, Thailand; 2 Department of Biotechnology, Faculty of Science, Mahidol University, Rama VI Road, Bangkok 10400, Thailand; 3 National Center for Genetic Engineering and Biotechnology (BIOTEC), Klong Luang, Pathumthani, 12120, Thailand; 4 Department of Anatomy, Faculty of Science, Mahidol University, Rama VI Road, Bangkok 10400, Thailand

## Abstract

**Background:**

From 2001-2003 monodon slow growth syndrome (MSGS) caused severe economic losses for Thai shrimp farmers who cultivated the native, giant tiger shrimp, and this led them to adopt exotic stocks of the domesticated whiteleg shrimp as the species of cultivation choice, despite the higher value of giant tiger shrimp. In 2008, newly discovered Laem-Singh virus (LSNV) was proposed as a necessary but insufficient cause of MSGS, and this stimulated the search for the additional component cause(s) of MSGS in the hope that discovery would lead to preventative measures that could revive cultivation of the higher value native shrimp species.

**Results:**

Using a universal shotgun cloning protocol, a novel RNA, integrase-containing element (ICE) was found in giant tiger shrimp from MSGS ponds (GenBank accession number FJ498866). *In situ *hybridization probes and RT-PCR tests revealed that ICE and Laem-Singh virus (LSNV) occurred together in lymphoid organs (LO) of shrimp from MSGS ponds but not in shrimp from normal ponds. Tissue homogenates of shrimp from MSGS ponds yielded a fraction that gave positive RT-PCR reactions for both ICE and LSNV and showed viral-like particles by transmission electron microscopy (TEM). Bioassays of this fraction with juvenile giant tiger shrimp resulted in retarded growth with gross signs of MSGS, and *in situ *hybridization assays revealed ICE and LSNV together in LO, eyes and gills. Viral-like particles similar to those seen in tissue extracts from natural infections were also seen by TEM.

**Conclusions:**

ICE and LSNV were found together only in shrimp from MSGS ponds and only in shrimp showing gross signs of MSGS after injection with a preparation containing ICE and LSNV. ICE was never found in the absence of LSNV although LSNV was sometimes found in normal shrimp in the absence of ICE. The results suggest that ICE and LSNV may act together as component causes of MSGS, but this cannot be proven conclusively without single and combined bioassays using purified preparations of both ICE and LSNV. Despite this ambiguity, it is recommended in the interim that ICE be added to the agents such as LSNV already listed for exclusion from domesticated stocks of the black tiger shrimp.

## Background

The term of monodon slow growth syndrome (MSGS) has been used by Thai shrimp farmers to refer to unusual retarded growth that has occurred in cultivated *P. monodon *since 2002. It has been suggested that an infectious agent may be the cause [1]. This contention was supported by the rapid spread of the problem and preliminary laboratory challenge tests showing that membrane-filtered (i.e., bacteria-free) tissue extracts from MSGS shrimp were able to cause MSGS when injected into *P. monodon *but not when injected into *P. vannamei *[2]. Thai researchers adopted a case definition to distinguish ponds exhibiting MSGS from ponds exhibiting slow growth caused by other problems [1]. According to this case definition, an MSGS pond must show a coefficient of size variation ≥35% together with freedom from hepatopancreatic parvovirus (HPV) (also called Penaeus monodon densovirus [3]) or any other severe hepatopancreas infection, plus any 3 of the following 5 characters: 1) unusually dark color, 2) average daily weight gain of less than 0.1 g/day at 4 months, 3) unusually bright yellow markings, 4) "bamboo-shaped" abdominal segments, and 5) brittle antennae. These features distinguish MSGS from stunted growth caused by monodon baculovirus (MBV) (also called Penaeus monodon polyhedrovirus [4]) or HPV [5,6].

An earlier survey revealed no correlation between the presence of several known shrimp pathogens [including infectious hypodermal and hematopoietic necrosis virus (IHHNV), also called Penaeus stylirostris densovirus [3]] and the problem of MSGS ponds [7]. The results suggested that a new pathogen might be the cause of MSGS. Separation of the purported agent from shrimp tissue homogenates has been difficult because of the presence of one or more known viruses and because bioassays require long incubation times of up to 1 month or more before results can be assessed. Therefore, we adopted a general "shot-gun" strategy to screen MSGS cases for the presence of unknown pathogens [1]. Briefly, it consisted of obtaining shrimp from MSGS case ponds followed by preparation of "viral" extracts for fractionation by gradient centrifugation. The resulting bands were removed and used for total nucleic acid extraction before shotgun cloning of cDNA prepared using random hexamer primers. Since total nucleic acid was used as the template, both RNA and DNA were amplified. Clones that hybridized with labelled, total DNA from normal *P. monodon *were eliminated and negative hybridization clones were sent for sequencing. Clones identified by Blast analysis to contain sequences of shrimp or previously screened pathogens were discarded, and remaining clones were used for *in situ *hybridization assays with shrimp from MSGS ponds and from normal growth ponds.

Using this strategy, one new RNA virus (LSNV) was identified [1] but its presence in shrimp was not initially correlated with the occurrence of MSGS ponds. However, subsequent work revealed that stunted shrimp from MSGS ponds gave strong *in situ *hybridization reactions for LSNV in retinal lesions that were not present in large shrimp from MSGS ponds or in shrimp from normal growth ponds that gave positive RT-PCR reactions for the presence of LSNV [8]. This led to the conclusion that LSNV was a necessary but not sufficient cause of MSGS and suggested that another factor(s) must be combined with LSNV to cause MSGS. One possibility for the additional factor was another pathogen(s). To examine this possibility, the shotgun strategy was used for further screening of shrimp from MSGS ponds. As a result, a unique clone was discovered that contained an integrase domain and that appeared to arise from RNA of viral-like particles present in tissue homogenates of shrimp from MSGS ponds.

## Results

### Density gradient separations from tissue homogenates

**Figure 1:**
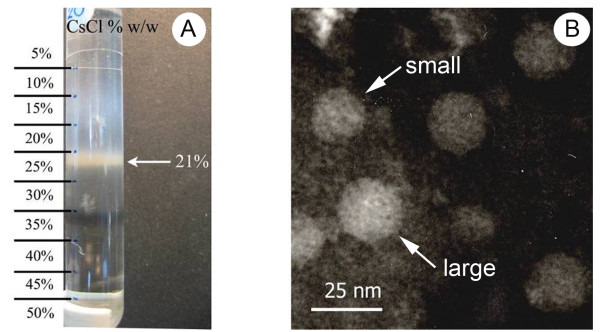
**Density gradient results**. (A) Photograph of 3rd-round gradient CsCl density gradient separation showing a band at 21% CsCl (1.1843 g/cm3). (B) Transmission electron micrograph (TEM) of a negatively-stained sample from the band in (A) showing viral-like particles of approximately 25 and 15 nm diameter (arrows) with some angular sides.

**Figure 2:**
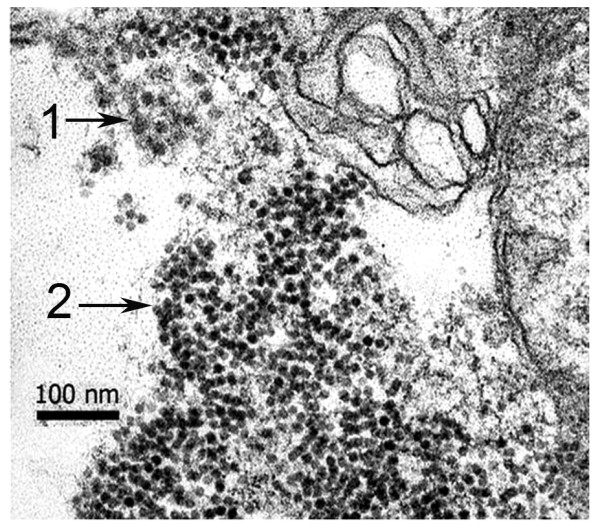
**TEM of naturally infected tissue**. Transmission electron micrograph of a lymphoid organ (LO) tissue section of *P. monodon *from an MSGS pond showing the presence of viral-like particles of approximately 25 nm diameter (arrow 1) and 15 nm in diameter (arrow 2).

Based on previous work suggesting that the causative agent(s) of MSGS were viral in nature [2], the gills of shrimp from MSGS ponds were subjected to homogenization and sucrose density gradient centrifugation that yielded several bands in the range of 10-50% (w/w) sucrose (not shown). Each band was collected separately and subjected to a second step of purification using 10-40% (w/w) CsCl gradients. Fractions of the single bands obtained from CsCl gradients were placed on coated grids and negatively stained with 2% phosphotungstic acid (PTA) at pH 7.4 for transmission electron microscopy (TEM). This process revealed that a band at 20-25% (w/w) CsCl gave presumptive viral-like particles. A third round of gradient separation yielded a distinct band at 21% CsCl (buoyant density 1.1843 g cm^-3^)[9] (Figure 1A). TEM of negatively-stained samples from this band revealed the presence of viral-like particles in two diameters of approximately 25 nm and 15 nm and with rounded to angular shapes indicating possible icosahedral morphology (Figure 1B). These were similar in size and shape to those seen by TEM in tissue sections of stunted shrimp from MSGS ponds (Figure 2).

### Shotgun cloning and screening for unknown, non-shrimp clones

**Figure 3:**
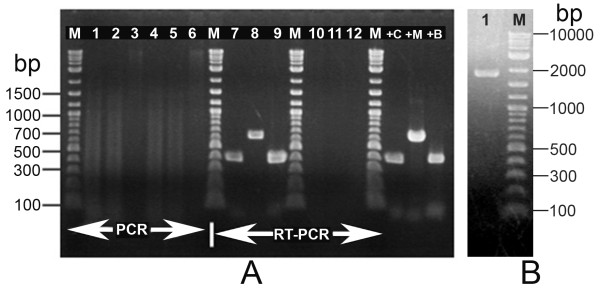
**PCR and RT-PCR results from density gradient band**. (A) Electrophoresis gel showing PCR and RT-PCR results using primers for Probe 1 (397 bp), Probe 2 (642 bp) and Probe 3 (399 bp) to carry out PCR and RT-PCR reactions with total nucleic acid template extracted from the 21% CsCl band. Lanes 1-6: PCR reactions carried out using template treated with DNase (Lanes 1-3) and RNase (Lanes 4-6) and showing none of the expected amplicons. Lanes 7-12: RT-PCR reactions (Probes 1-3 sequentially) carried out using template treated with DNase (Lanes 7-9) and RNase (Lanes 10-12) and showing amplicons with DNase treatment but not RNase treatment, indicating that the ICE amplicons originated from RNA. Lanes +C, +M and +B = Positive controls for Probes 1 to 3, respectively. Lanes M = DNA ladder marker. (B) Electrophoresis gel showing the predicted 1958 bp amplicon obtained using the forward primer of Probe 1 and the reverse primer of Probe 3.

**Figure 4:**
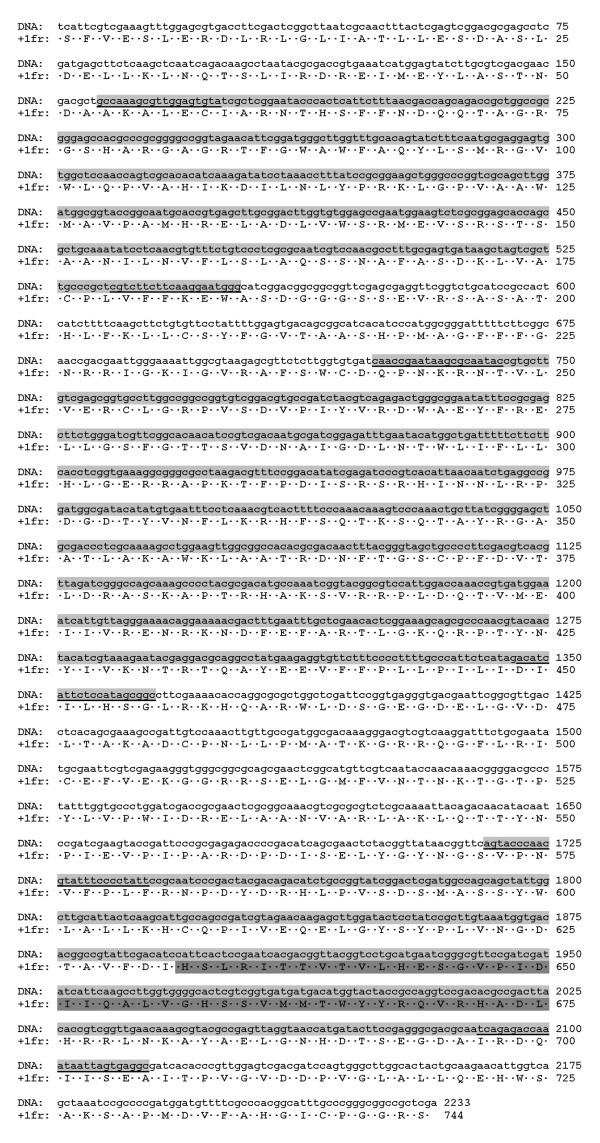
**ICE sequence**. Putative open reading frame of 2233 nucleotides in ICE (GenBank accession number FJ498866) corresponding to an uninterrupted sequence of 744 deduced amino acids by +1 frame translation. A putative phage integrase domain is outlined in dark grey in the deduced amino acid sequence. The three probe sequences (Probe 1 of 397 bp; Probe 2 of 642 bp; Probe 3 of 399 bp) are outlined sequentially in light grey with their primer sequences underlined. A single fragment of 1958 bp could also be amplified using the forward primer of Probe 1 and the reverse primer of Probe 3.

**Figure 5:**
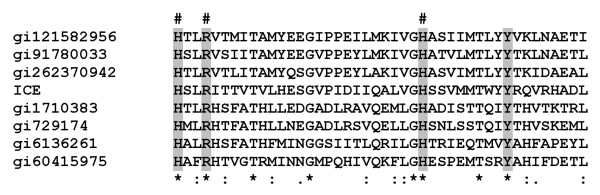
**Alignment of ICE integrase domain**. CLUSTAL 2.0.12 alignment of DNA cutting enzyme domains of GenBank records most similar to that of ICE. The highly conserved histidine-arginine-histidine (H-R-H) sequence motif (#) of the integrase family catalytic traid plus a nearby tyrosine (Y) nucleophile are outlined in grey. gi121582956 = *Polaromonas *phage integrase; gi91780033 = *Burkholderia *phage integrase; gi262370942 = *Acinetobacter *phage integrase; gi1710383 = *Bacillus subtilis *tyrosine recombinase; gi729174 = *Bacillus subtilis *tyrosine recombinase; gi6136261 = *Enterobacteria *phage P2 integrase; gi60415975 = *Staphylococcus aureus *transposase B.

From a total of 180 clones obtained from shotgun cloning of the nucleic acid extract from the 21% CsCl density gradient band, 31 did not hybridize with labeled DNA from normal shrimp in dot-blot hybridization assays, and these were selected for nucleic acid sequencing. Partial sequencing from both ends revealed that 10 clones contained shrimp sequences and 4 bacterial sequences. These clones were not studied further. Of the 17 remaining clones, CLUSTAL W analysis of insert end-sequences revealed that all contained common sequence regions. Thus, 2 of the largest inserts of approximately 2.2 kb called B004 (2250 bp) and C010 (2223 bp) were subjected to single-pass sequencing. This revealed that the sequence of C010 fell completely within the sequence of B004. Primers were designed based on the consensus sequence for C010/B004 to produce amplicons of 397 bp from the 5' end (Probe 1), 642 bp from the middle (Probe 2) and 399 bp from the 3' end (Probe 3) (Table 1) of the sequence using PCR and RT-PCR amplification with DNase-treated and RNase-treated total nucleic acid extracts from the 21% CsCl band. The expected amplicons were obtained only by RT-PCR from the DNase-treated RNA extract (Figure 3A). An additional RT-PCR reaction was carried out using the forward primer of Probe 1 and the reverse primer of Probe 3 to obtain a predicted amplicon of 1958 bp (Figure 3B) that was cloned and sequenced from both strands. All the data was combined to obtain the final consensus sequence of 2233 bp shown in Figure 4 (GenBank accession number FJ498866). This was subjected to a Blastn search that revealed no significant identity to known nucleic acid sequences except for 100% identity in 46 bases near the 3' end of the sequence with thrombospondin of *Penaeus (Marsupenaeus) japonicas *(GenBank AB121211). A frame +1 translation of the 2233 bp sequence yielded a single uninterrupted sequence of 744 deduced amino acids that had no known homology to any protein or translated protein sequence at GenBank except for an integrase protein of bacteriophage SH046 of *Acinetobacter johnsonii *(E value 1 × e^-4^) near the 3' end, but at low identity (29%). The deduced amino acids at positions 1894-2025 (44 amino acids) in the C-terminal portion of the sequence had homology to a conserved domain for phage integrases and DNA breaking enzymes in the DNA_BRE_C family, as outlined in grey in Figure 4 and shown in alignment with similar sequences in Figure 5. Thus, for convenience, the sequence in Figure 4 was called an integrase containing element (ICE). The acronym ICE as used herein should not be confused with that used for "integrating and conjugative elements (ICEs)" in bacteria [10]. Our several attempts to extend the ICE sequence by either 3' or 5' RACE failed.

**Table 1:** RT-PCR primers used to amplify the portions of the ICE sequence

Primer names	**Sequence 5**'**-3**'	Melting temperature (Tm, °C)	Expected product (bp)
Probe 1-Fr	**GCCAAAGCGTTGGAGTGTA**	57.56	397
Probe1-Rv	**CCCATTCCTTGAAGAAGACG**	57.80	

Probe 2-Fr	**CAACCGAATAAGCGCAATAC**	60.40	642
Probe 2-Rv	**GCCGCTATGGAGAATGATGT**	58.40	

Probe 3-Fr	**AGTACCCAACGTATTTCCCCTATT**	58.55	399
Probe 3-Rv	**GCCTCACTAATTATTTGGTCTCTGA**	58.70	

LSNV-Fr LSNV-Rv	**CGTTCGCTTCTCCCGAGTGGT TTGCCCCAGAAACGTATTGGCA**	60 61	600

**Nested primers**			

Probe 3-NFr	**AGCAGCTATTGGCTTGCATT**	60	204
Probe 3-NRv	**TGTCATCATCACCGACGAGT**	60	

LSNV-NFr	**TTGCCTTCTCCCGAGTGGTC**	64	195
LSNV-NRv	**CCGGCTGAGGTAGCTGCTTG**	66	

### LSNV also in the 21% CsCl gradient band

Total nucleic acid extracts from the 21% CsCl gradient band also gave positive RT-PCR reactions for LSNV. Attempts to remove LSNV reactivity by further density gradient centrifugation failed. Therefore, it is important to understand that the inocula used for all challenge tests consisted of a mixture of at least LSNV and ICE. In addition to LSNV, the extract was tested and found negative for IHHNV by PCR.

### Northern blot of RNA from the 21% CsCl gradient band

When RNA extracted from the 21% CsCl gradient band was subjected to northern blot analysis using ICE probe 3, a positive hybridization signal at approximately 3.0 kb was obtained for ICE, while no signal was detected in the RNA extracted from SPF shrimp (Additional file 1).

### PCR screening for ICE and LSNV in shrimp from MSGS ponds

When one-step RT-PCR protocols for ICE Probe 3 and for LSNV were used with template RNA extracted from gills of shrimp from ponds that fit the MSGS case definition, it was revealed that most of the shrimp tested gave positive reactions for both LSNV and ICE (Table 2). By contrast, domesticated *P. monodon *specimens in families with no history of association with MSGS and shrimp from normal growth ponds were always negative for ICE even though some were positive for LSNV (not shown).

**Table 2:** One-step RT-PCR results for ICE and LSNV from MSGS ponds

Sample Set #	Location/date	% CV	Samples	LSNV	ICE Probe 3
1	CP farm, Laem-Singh district Chanthaburi/ 20 Aug 2004	46%	10	+10	+10

2	Khun Mug farm, Laem-Singh district Chanthaburi/ 28 Jul 2005	37%	10	+7	+6

3	Pakpranung district, Nakornsrithammarat 10 Apr 2006	40%	10	+8	+8

4	Vipath farm, Sichol district, Nakornsrithammarat/ 10 Apr 2006	42%	10	+10	+7

5	Ko-Heng fram, Kantrung district Trung 10 Apr 2006	45%	10	+10	+8

6	Farm Laem-Singh district, Chanthaburi 5 Oct. 2006	38%	6	+3	+2

### *In situ *hybridization tests for ICE and LSNV in shrimp from MSGS ponds

**Figure 6:**
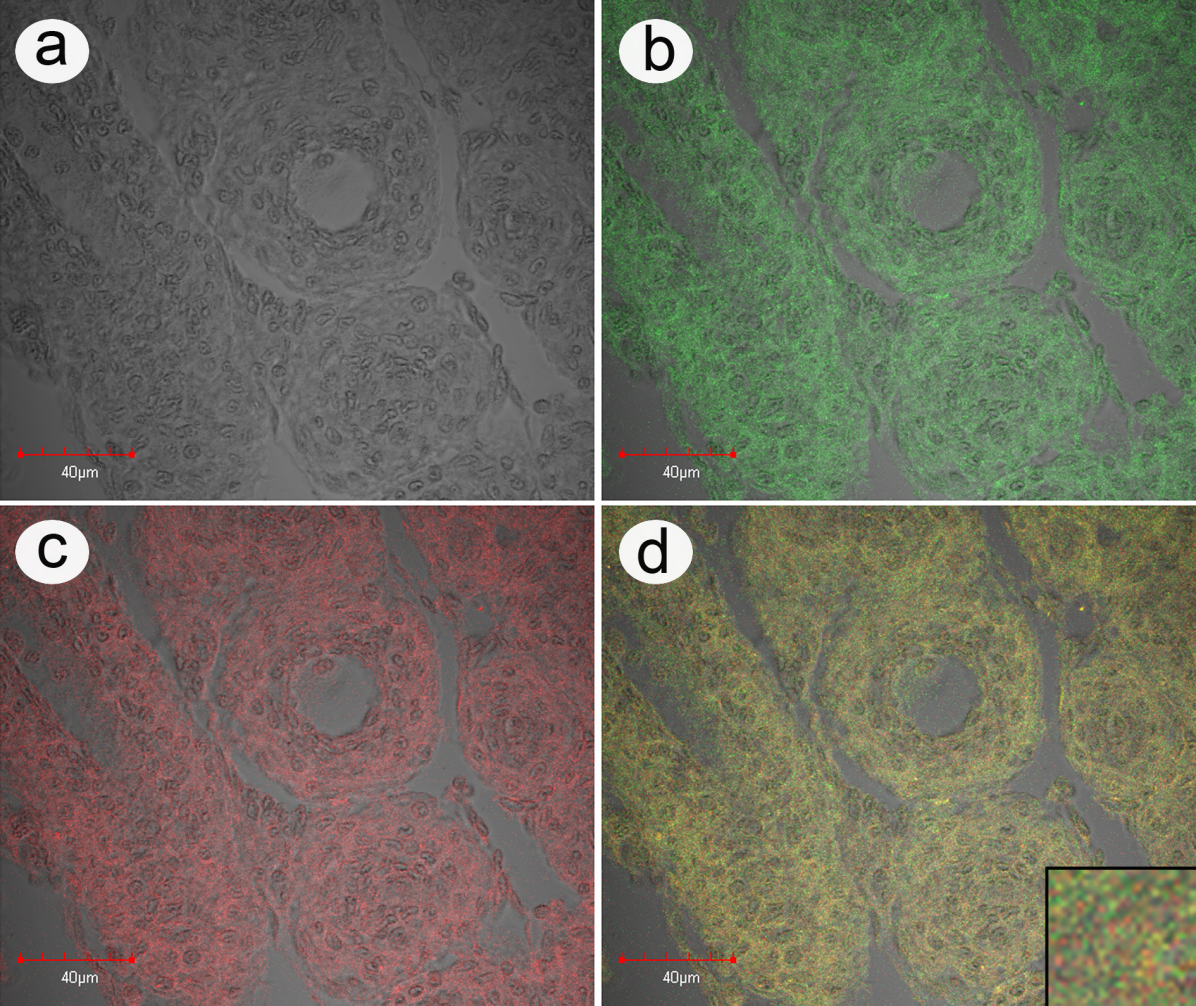
***In situ *hybridization with natural MSGS specimen**. Example of an *in situ *hybridization reaction using a rhodamine-labeled ICE probe (red) together with an FITC-labeled LSNV probe (green) with lymphoid organ (LO) tissue of a *P. monodon *specimen from an MSGS pond that was positive by RT-PCR for both ICE and LSNV. a) Phase contrast image of tissue; b) Image of LSNV fluorescence; c) Image of ICE fluorescence; d) Combined images of a to c with a magnified insert showing that the fluorescence distribution for the two signals is single (green or red) and combined (yellow).

Using a rhodamine-labeled probe (red fluorescence) prepared from ICE and a fluorescence probe (green fluorescence) prepared from a fragment of LSNV for *in situ *hybridization tests with lymphoid organ (LO) tissue of shrimp from MSGS ponds resulted in positive results for both LSNV and ICE (Figure 6).

### Viral-like particles in LO tissue positive for ICE and LSNV by *in situ *hybridization

When parallel tissue samples from shrimp showing positive hybridization with ICE probes were subjected to examination by transmission electron microscopy (TEM), non-enveloped, icosahedral viral-like particles approximately 25 nm and 15 nm diameter were seen, similar to those previously shown for samples from MSGS ponds (Figure 2).

### Shrimp challenge tests using inoculum from the 21% CsCl gradient band

In an attempt to fulfil Koch's postulates as modified by Rivers (1937), two challenge tests were carried out. In the first challenge, primary inoculum was prepared from the original 21% CsCl gradient band and injected into juvenile *P. monodon *that were maintained for 60 days before analysis and also for preparation of inoculum (using the same protocol used to prepare the primary inoculum) for a second challenge test. In the second challenge test, the secondary inoculum was injected into a second set of test shrimp, also maintained for 60 days before analysis. The size of the domesticated shrimp used for the first challenge was 5.132 ± 0.767 g while that for the second was 5.633 ± 0.798 g. Some of the stock shrimp were positive for LSNV by RT-PCR (i.e., 15% of the first challenge stock and 20% of the second challenge stock).

At the end of 60 days in the first challenge experiment, there was no significant difference in survival between the test (61.4%) and control (64.3%) groups. One-step or nested RT-PCR (Table 3) revealed that all test and many control shrimp (50%) were positive for LSNV by nested RT-PCR, although the overall LSNV reactions tended to be more prevalent (100% vs 50-60%) and stronger (i.e., more 1-step PCR positive) than those from the control shrimp when using the same amount of total RNA template. By contrast, only the test shrimp were positive for ICE by either one-step or nested RT-PCR. With respect to other parameters, there were no significant differences (p > 0.05) in mean length, weight or survival between the test and control shrimp although the means for weight and length in the test group were lower than those in the control groups (Additional file 2) and the statistics software package advised caution in interpreting the negative t-test results because the power for the tests was low (i.e., 0.05 to 0.01 compared to the desired value of 0.8). In addition, the test group showed a greater tendency for abnormally dark coloration and brittle antennae than did the control group.

**Table 3:** RT-PCR results for ICE and LSNV from bioassay shrimp

						LSNV RT-PCR				ICE RT-PCR		
								
Experiment		Group		No. Samples		One-step		Nested		One-step		Nested
1^st ^challenge		Control		10		+1		+5		-		-
		Test injection		10		+4		+10		+2		+9

2^nd ^challenge		Control		10		+2		+6		-		-
		Test injection		10		+6		+10		+2		+7

**Figure 7:**
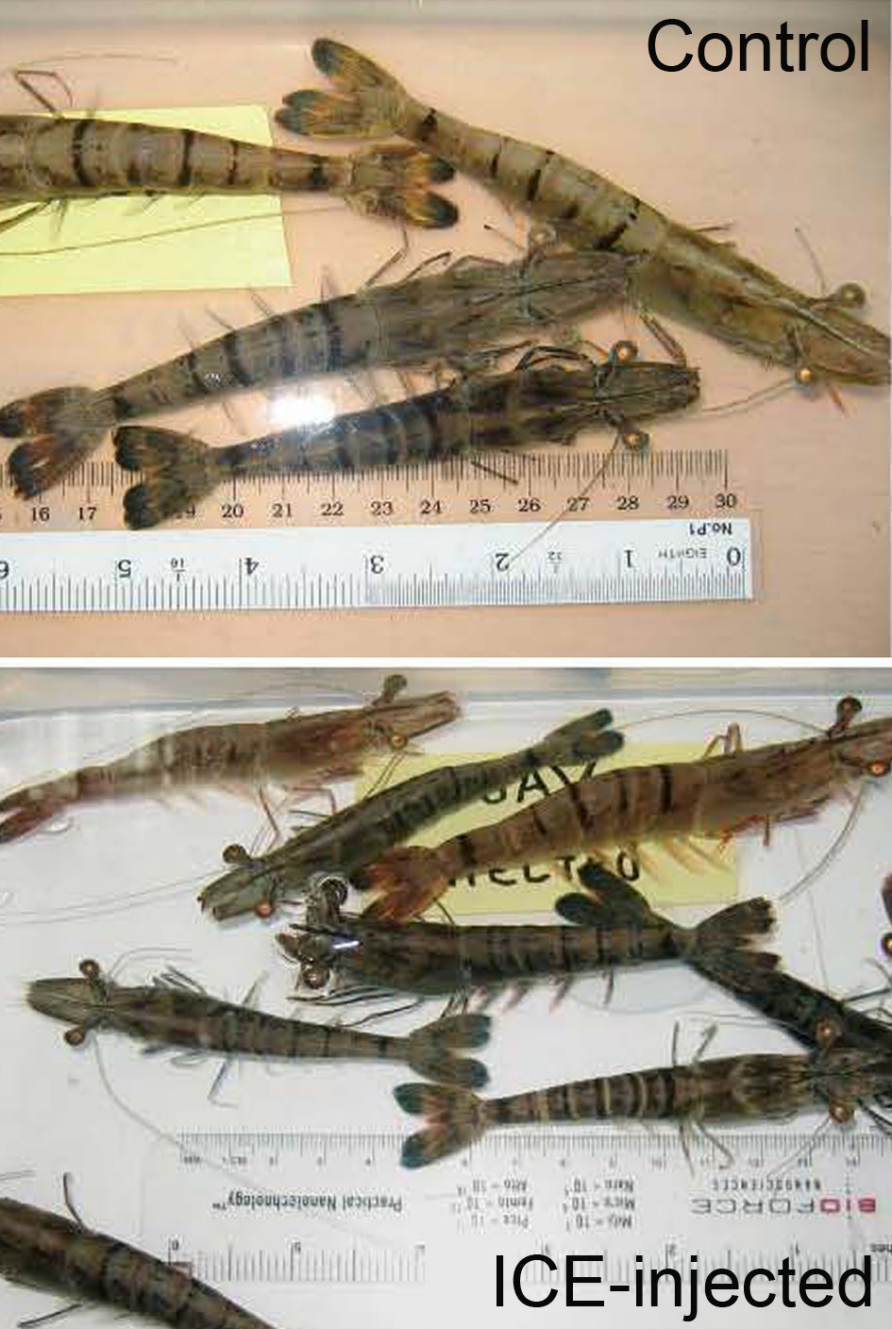
**Gross signs of MSGS in bioassay shrimp**. Comparison at the same scale of gross morphology of control and ICE-injected *P. monodon *from the second challenge test. The ICE-injected shrimp are generally smaller and darker colored than the control shrimp.

At the end of 60 days in the second challenge experiment, there was again no significant difference in survival between the test (75.0%) and control (78.6%) groups. As with the first test, there was a tendency for abnormally dark coloration and brittle antennae in the test but not the control group (Figure 7). Also, one-step or nested RT-PCR (Table 3) revealed that all of the test shrimp and many of the control shrimp were positive for LSNV by nested RT-PCR, while only the test shrimp were positive for ICE by either one-step or nested RT-PCR (as in the first challenge experiment). However, in the second challenge experiment, the test group had significantly lower mean weight (p = 0.001), weight gain (p = 0.032), length (p = 0.001) and length gain (p = 0.001) than the control group (Table 4).

**Table 4:** Shrimp survival and growth from the 2^nd ^bioassay

		Control TN Buffer		ICE Injection	
		
Replication	Parameters	1 Day	60 Days	1 Day	60 Days
	n	7	6	7	5
1	mean of body weight (g)	5.7 ± 0.8	8.7 ± 1.0	5.3 ± 0.5	6.6 ± 1.0
	mean of length (cm)	5.5 ± 0.3	7.9 ± 0.4	5.4 ± 0.3	7.2 ± 0.6

	n	7	5	7	5
2	mean of body weight (g)	5.9 ± 0.9	8.8 ± 0.9	5.5 ± 0.9	7.1 ± 1.1
	mean of length (cm)	5.4 ± 0.4	8.0 ± 0.8	5.5 ± 0.4	7.3 ± 0.8

	n	7	5	7	6
3	mean of body weight (g)	5.7 ± 0.8	10.3 ± 2.1	6.2 ± 0.7	6.9 ± 0.7
	mean of length (cm)	5.4 ± 0.3	8.0 ± 0.8	5.7 ± 0.3	7.5 ± 0.4

	n	7	5	7	6
4	mean of body weight (g)	5.1 ± 0.6	10.3 ± 1.9	5.7 ± 1.0	6.4 ± 0.6
	mean of length (cm)	5.4 ± 0.3	8.2 ± 1.0	5.4 ± 0.3	7.2 ± 0.4

Means of means ± SE	mean of body weight (g)	5.6 ± 0.3	9.5 ± 0.8*	5.7 ± 0.3	6.8 ± 0.3*
	mean of length (cm)	5.4 ± 0.04	8.0 ± 0.1*	5.5 ± 0.1	7.3 ± 0.1*

% mortality		25.0		21.4	

For totals, the numbers with asterisks in each row were significantly different (p = 0.001 to 0.032, see text for details).

### *In situ *hybridization tests with experimentally challenged shrimp

**Figure 8:**
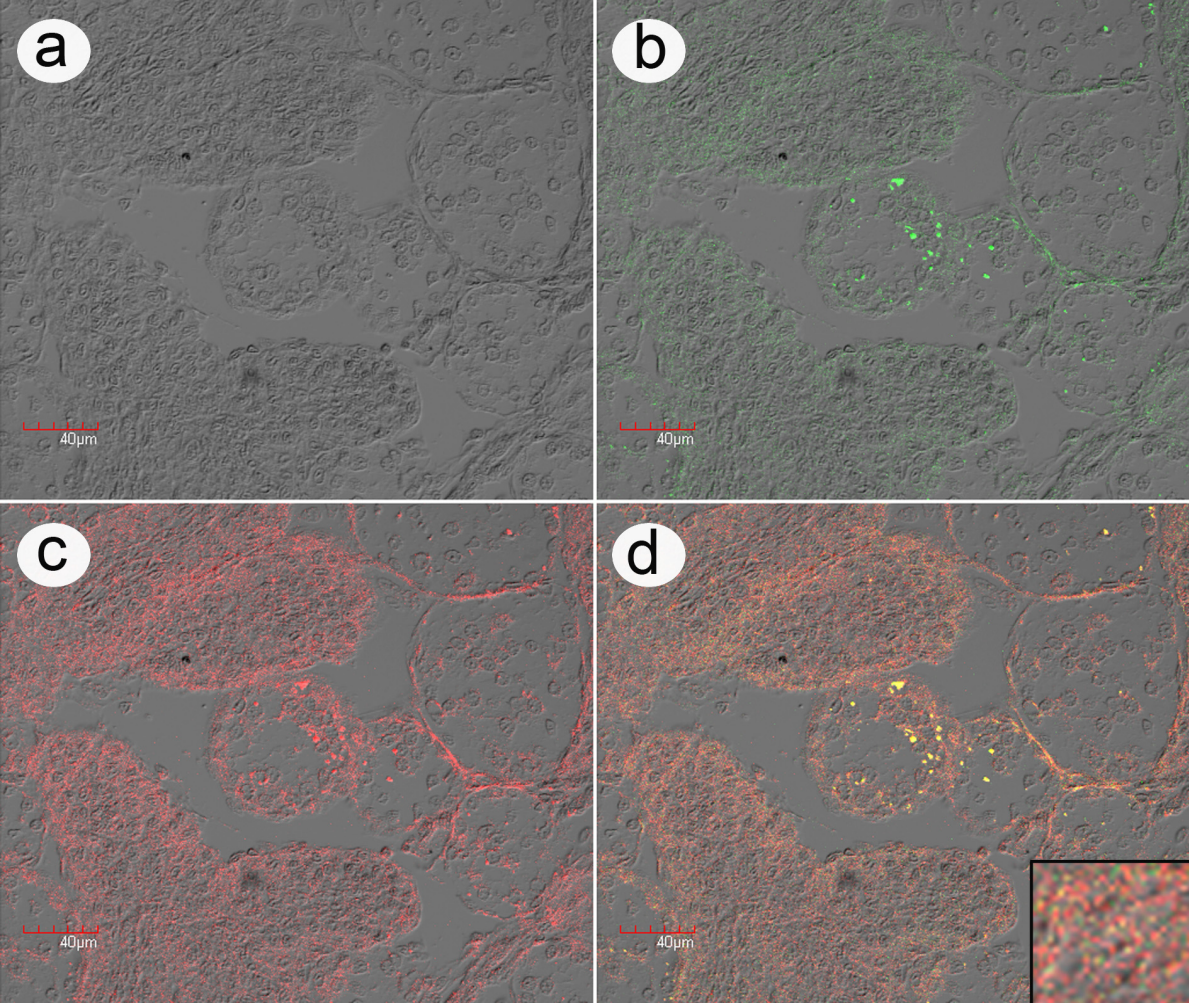
**High magnification *in situ *hybridization of LO from bioassay**. High magnification example of an *in situ *hybridization reaction using a rhodamine-labeled ICE probe (red) together with an FITC-labeled LSNV probe (green) with lymphoid organ (LO) tissue of a *P. monodon *specimen from challenge test 1 that was positive by RT-PCR for both ICE and LSNV. This is a photomicrograph of a single confocal microscope image layer showing that the fluorescence distribution for ICE and LSNV is in the cell cytoplasm and not the nuclei. A lower magnification photomicrograph showing brighter fluorescence from multiple confocal microscope image layers is shown in Additional file 4. a) Phase contrast image; b) Image of LSNV fluorescence; c) Image of ICE fluorescence; d) Combined images with a magnified insert showing that the fluorescence distribution for the two signals is single (green or red) and combined (yellow). Note that the fluorescence is located in the cell cytoplasm and is most intense in the normal tubules of the LO and much less intense in the spheroids (i.e., the circular groups of cells surrounded by a ring of sheath cells that are most clearly visible in the combined image d). Negative control images from a normal shrimp specimen are shown in Additional file 3.

**Figure 9:**
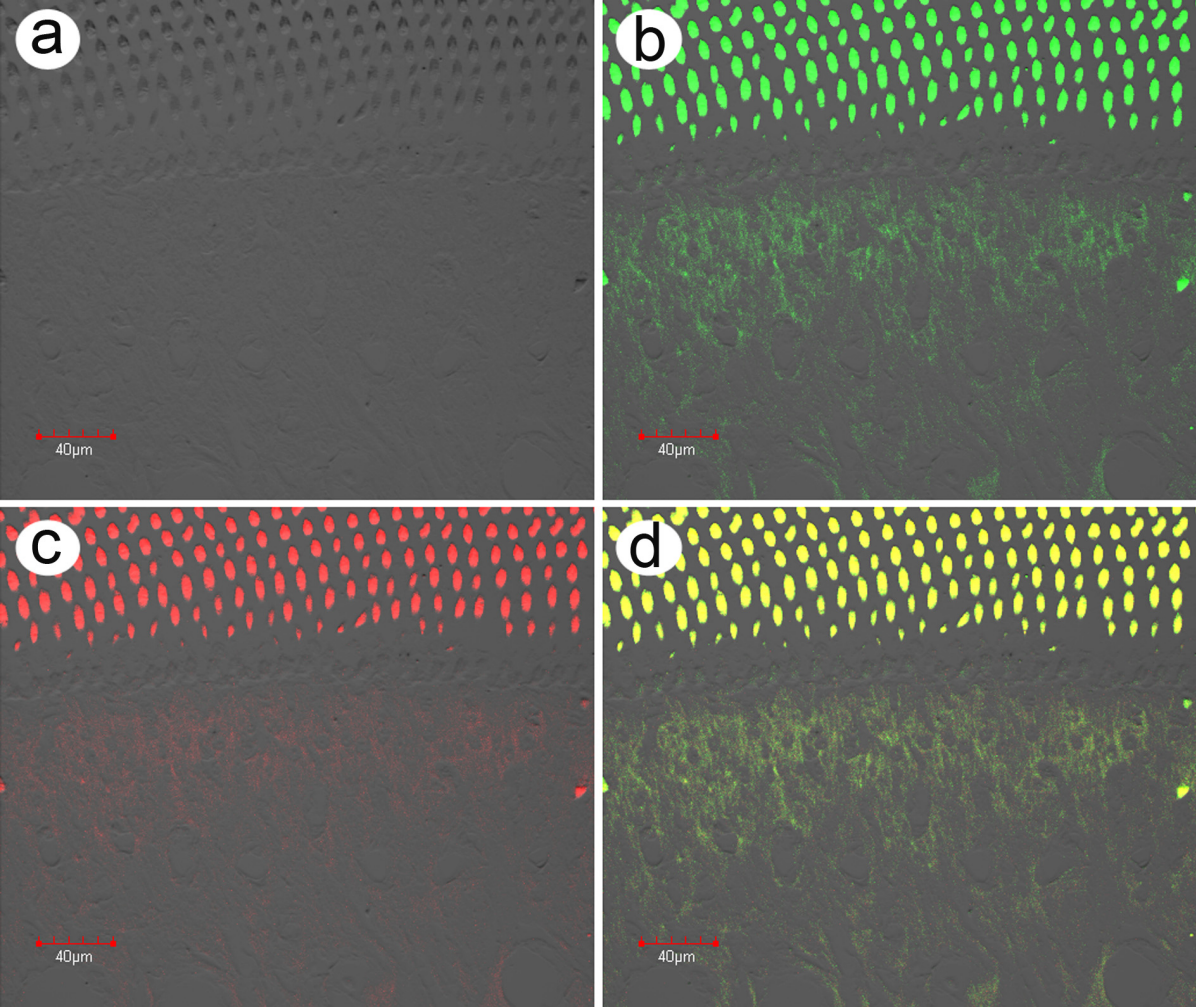
***In situ *hybridization of eye from bioassay**. Example of confocal photomictographs of the fasciculated zone of the eye of a challenged shrimp specimen from test 2, postive for both LSNV and ICE by RT-PCR. a) Phase image; b) Image showing positive fluorescence for LSNV in the fasciculated zone under the retinal layer that is below the false-positive fluorescence of the crystalline tracts; c) Image showing positive fluorescence for ICE in the fasciculated zone with false-positive fluorescence as in image (b). d) Combined images showing co-localized positive fluorescence (yellow) for ICE and LSNV in the fasciculated zone and false positive fluorescence in the crystalline tracts. Negative control images are shown in Additional file 5.

**Figure 10:**
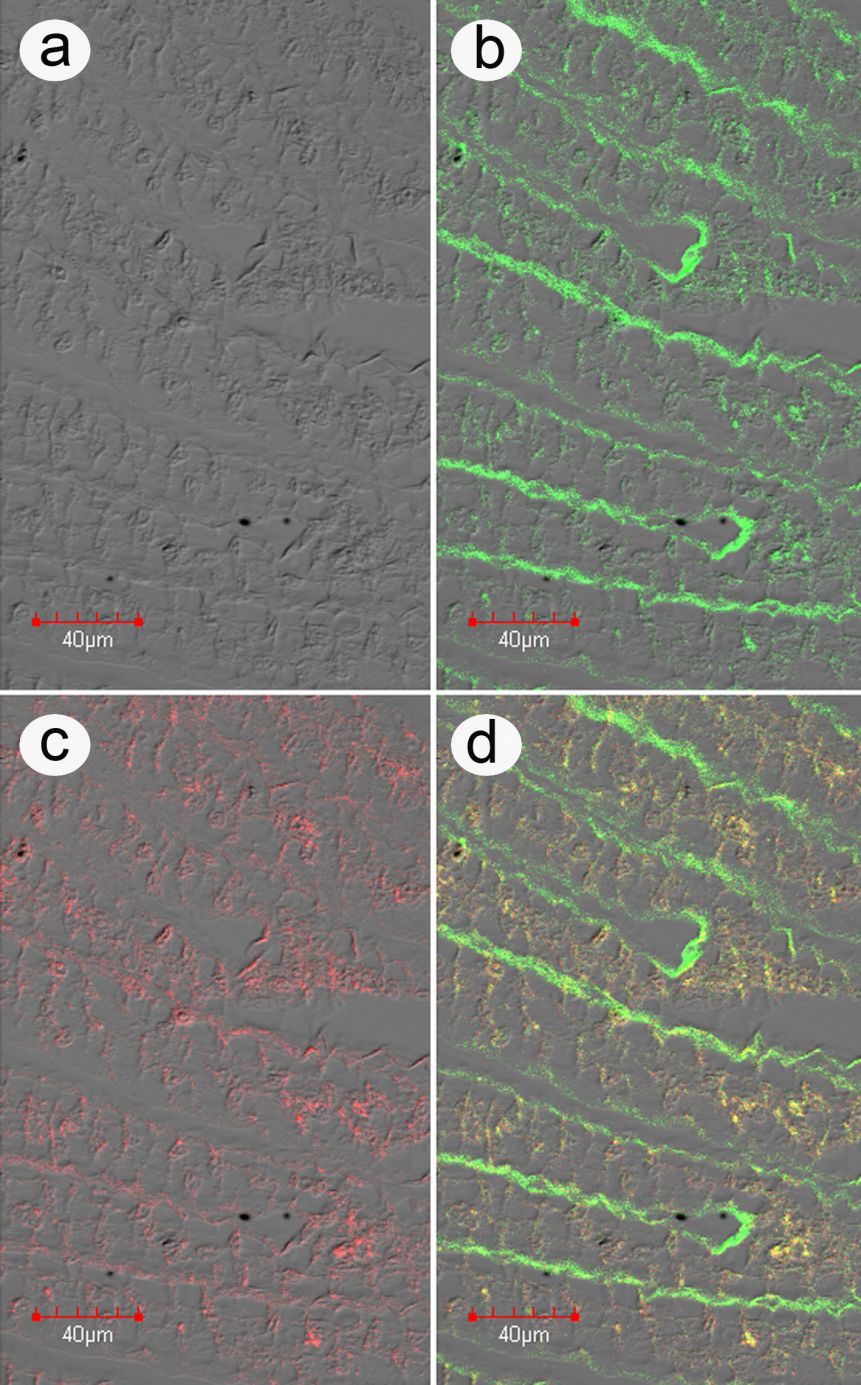
**In situ hybridization of gills from bioassay**. Example of confocal photomictographs of gill tissue from a challenged shrimp specimen from test 2, postive for both LSNV and ICE by RT-PCR. a) Phase image; b) Image showing positive fluorescence for LSNV in the pillar cells and false-positive fluorescence of the cuticle; c) Image showing positive fluorescence for ICE in the pillar cells and false-positive fluorescence of the cuticle. d) Combined images showing co-localized positive fluorescence (yellow) for ICE and LSNV in the pillar cells and false positive fluorescence in the cuticle.

An arbitrary selection of 5 shrimp each from the control and test groups from the second challenge experiment was subjected to *in situ *hybridization assays for ICE and LSNV. Because of the previously described link between LSNV retinopathy and MSGS, the assays focused on tissues of the eyes and eyestalks in addition to the LO and gills. Results for the LO and gills showed that control shrimp positive for LSNV by RT-PCR gave positive hybridization results in the cell cytoplasm for LSNV only (not shown), while those negative by RT-PCR gave negative hybridization results for both ICE and LSNV (Additional file 3). By contrast, the test shrimp gave positive hybridization results in the cell cytoplasm of LO cells for both ICE and LSNV (Figure 8 and Additional file 4). The fluorescence for both probes was evenly distributed in the cytoplasm, did not completely overlap and was most intense in normal LO tubules, as opposed to LO spheroids. The eyes of both test and control shrimp showed no retinopathy, but those of the control shrimp that tested either negative or positive for LSNV were negative for both LSNV and ICE (Additional file 5) while test shrimp showed positive *in situ *hybridization reactions for both ICE and LSNV (Figure 9). These results supported the previously described link between retinopathy and LSNV [8]. In addition to the LO and eyes, gills of the test shrimp only were also positive for both ICE and LSNV (Figure 10).

## Discussion

### The nature of ICE

Here we have shown the presence of an integrase containing element (ICE) that is located together with Laem-Singh virus (LSNV) in the cytoplasm of cells of the lymphoid organ (LO), gills and the sub-retinal, fasciculated zone of the eyes of black tiger shrimp exhibiting gross signs of disease referred to as monodon slow growth syndrome (MSGS). Since ICE was isolated from a density gradient band derived from gill tissue homogenates of shrimp from an MSGS pond and since the gradient band contained two sizes of viral-like particles (approximately 25 and 15 nm diameter) it is possible that ICE originated from these particles. Since the extracts containing ICE also contained genomic material from LSNV, and since the diameter of unenveloped LSNV particles has been estimated at approximately 27 nm from thin tissue sections [1], it is reasonable to suggest that ICE may have originated from the particles 15 nm in diameter. If so, the positive RT-PCR reactions indicated that the particles contained the ICE fragment in the form of RNA. Although this is reminiscent of extra small virus (XSV) that has been described from the giant freshwater prawn *Macrobrachium rosenbergii *[11] as a possible satellite virus [12,13], the ICE sequence shared no significant identity to the XSV genome sequence or any other known satellite virus sequence. Nor does the XSV genome or other known satellite genomes contain integrase genes [14]. Similarly, the ICE sequence showed no significant homology to the genomes of unenveloped RNA viruses with integrase genes that can be found in the families *Pseudoviridae *[15] and *Metaviridae *[16] that are related to enveloped viruses in the family *Retroviridae *[17]. In the *Pseudoviridae *and *Metaviridae *viral-like particles are sometimes produced in insect hosts, but the ones reported are much larger than 15 nm. In addition, phylogenetic comparisons among the reverse-transcribing viruses are usually based on their reverse transcriptase (RT) sequences and the lack of an RT sequence from ICE made such a comparison impossible. ICE also contained no group-specific antigen-like (*gag*-like) sequence for structural proteins, another common feature of viral-like members of the families *Pseudoviridae *and *Metaviridae*. If the problem of unsuccessful 3' and 5' RACE reactions could be resolved with ICE, RT and *gag *sequences might be revealed and allow standard phylogenetic comparisons. On the other hand, the northern blot result indicated that the full size of the RNA fragment containing the ICE target sequence was not a great deal larger than the ICE clone raising the question as to whether it would be sufficiently large to contain RT and *gag *sequences. Although ICE clearly contained an integrase domain with a conserved catalytic triad H-R-H and a conserved tyrosine (Y) in a binding region that may be involved in host genome insertion [18,19], the 44 amino-acid integrase domain showed homology only to integrases or recombinases of bacteria and bacteriophages and not viral-like members of the families *Pseudoviridae *and *Metaviridae*. This is also problematic. One might ask whether the ICE sequence arose from bacteria in the shrimp homogenates. We believe this to be unlikely since inspection by light microscopy and TEM revealed no bacteria in the parallel tissue sections that were positive for ICE by *in situ *hybridization. Thus, the nature of ICE and its phylogenetic relationship to other viral-like entities known to occur in invertebrates is an enigma.

### Association of ICE with LSNV and MSGS

Despite the uncertainty regarding the nature and phylogenetic relationships of ICE, we have shown that there is a strong tendency for it to be associated with LSNV in shrimp samples from MSGS ponds, and in shrimp challenged with inoculum from a density gradient band derived from tissue homogenates of shrimp from MSGS ponds. Furthermore, *in situ *hybridization analysis revealed that ICE and LSNV occur together in LO and eyestalk tissues previously shown to give positive reactions for LSNV in stunted shrimp from MSGS ponds (Pratoomthai et al., 2008). In no case were shrimp found that were positive for ICE in the absence of LSNV. Since LSNV was proposed to be a necessary but insufficient cause of MSGS based on its association with retinal lesions only in small shrimp from MSGS ponds, the co-association of ICE with LSNV in such lesions suggests that it may also be causally linked to MSGS. Although the inability to clearly separate ICE from LSNV for laboratory challenge tests makes it difficult to prove this causal relationship, our results using inoculum positive for ICE and LSNV to challenge shrimp already positive for LSNV did support the argument that they may be co-causes of MSGS. This is because the control shrimp already infected with LSNV did not show retarded growth, darkening or *in situ *positive retinal tissues that were seen in the challenged shrimp, even though the LO of the control shrimp were positive for LSNV. It is true that the ICE and LSNV positive reactions in the test shrimp were not associated with retinal lesions in our challenge tests, but this may have been due to the age of the shrimp at the time of challenge or other experimental parameters that differed from those in shrimp ponds. In addition, it cannot be ruled out that the inoculum contained yet another viral-like agent(s) that was not identified but moved together with LSNV and ICE. Thus, final proof that LSNV and ICE are causally associated with MSGS will probably have to await the advent of infectious clones of ICE and LSNV so that perfectly controlled single and dual challenge tests can be carried out. Unfortunately, complete sequences of neither ICE nor LSNV are yet available.

## Conclusions

An ICE clone was derived from a process of differential centrifugation that would have eliminated bacteria from the final CsCl gradient, and the gradient band contained no bacterial cells. The nucleic acid clone derived from that band had no sequence identity to any known bacterium, and PCR and RT-PCR tests with that nucleic acid revealed that the clone arose from RNA, not DNA. Further, an *in situ *hybridization probe derived from that sequence gave positive hybridization results in the cytoplasm of cells of the lymphoid organ and eyes of shrimp where no bacterial cells were seen by either light microscopy or by electron microscopy, but where viral-like particles were seen by electron microscopy. Finally, the inoculum for the challenge trials was prepared with ultrafiltration steps that would have removed bacterial cells. Altogether, we may conclude that the ICE element is not from a bacterium, but that its nature remains elusive. Despite this ambiguity, we found that ICE occurred together with LSNV only in shrimp from MSGS ponds and only in shrimp showing gross signs of MSGS after injection with a preparation containing ICE and LSNV. ICE was never found in the absence of LSNV although LSNV was sometimes found in normal shrimp in the absence of ICE. This association between ICE and LSNV suggests that they may act together as component causes of MSGS. However, conclusive proof that they cause MSGS is not possible without single and combined bioassays using purified preparations of both ICE and LSNV.

Since the discovery of LSNV in 2006, it has been added to the list of viruses to be excluded from domesticated, specific pathogen free (SPF) stocks of *Penaeus monodon *in Thailand and it has been recommended that shrimp farmers avoid stocking post larvae RT-PCR positive for LSNV to avoid MSGS. In the same way, we recommend that ICE be added to the list of excludable agents in development of SPF stocks, since it is not highly prevalent in wild stocks of *P. monodon *that might be sourced as founders for such stocks. In addition, the availability of RT-PCR methods makes it possible to carry out continual monitoring in research programs to determine whether there is a positive correlation between MSGS and simultaneous presence of LSNV and ICE in cultivated *P. monodon*.

## Methods

### Natural and experimental shrimp for viral isolation and histology

Live shrimp from a shrimp pond that fit the MSGS case definition were obtained from Laem-Singh district Chanthaburi province, on the eastern coast of Thailand on 20^th ^August 2004. The mean shrimp weight was approximately 13 g (i.e., 77 shrimp per kg) and the CV was 46% CV at 120 days cultivation (average daily weight gain of 0.1 g/day). A total of 5 kg were collected. Most of the gills were removed from the living shrimp for immediate homogenization and gradient centrifugation, but some were fixed, together with the remaining body for normal histological examination by light microscopy [20]. Other, small samples were fixed for transmission electron microscopy [1].

### Transmission electron microscopy

Natural or experimental shrimp were stunned in ice water, dried with a towel and surface sterilized using 70% ethanol before aseptic dissection and removal of tissues for cutting into small pieces and washing with 0.15 M Millonig's phosphate buffer (40 mM NaH_2_PO_4_, 100 mM Na_2_HPO_4_, 170 mM NaCl, 15 mM sucrose, pH 7.4). The pieces were prefixed with 4% glutaraldehyde in 0.15 M Millonig's buffer at 4°C overnight, and then post-fixed in 1% OsO_4 _in the same buffer at 4°C for 1 h prior to being processed routinely for conventional embedding in Spurr's resin. Ultrathin sections were stained with uranyl acetate and lead citrate before viewing with a Hitachi H-500 transmission electron microscope at 75 kV.

### Inoculum stock preparation

Pooled gills were collected from natural or experimental living shrimp and stored in separate cryotubes under liquid nitrogen. Following rapid thawing, gill samples were homogenized in TN buffer (0.02 M Tris-HCl, 0.4 M NaCl, pH 7.4) using a glass homogenizer. The resulting suspension was clarified 3 times by first-round centrifugation at 600 *g *for 10 min at 4°C. The first pellet obtained was resuspended in TN buffer, re-homogenized, and centrifuged for 15 min at 3,500 g. The second pellet was re-homogenized and centrifuged for 30 min at 16,000 *g*. The 3 resulting supernatants were pooled and the whole was ultracentrifuged at 220,000 *g *for 2 h. The pellet obtained was resuspended after overnight incubation in TN buffer and layered on a 10 to 50% (w/w), sucrose gradient for centrifugation at 200,000 *g *for 6 h.

Each fraction containing presumptive viral particles was diluted in TN buffer and re-centrifuged at 300,000 *g *for 2 h before the pellet was re-suspended again after overnight incubation in TN buffer. The final purification was performed by layering the pellet fraction onto a 10 to 40% (w/w) CsCl gradient and centrifuging at 200,000 *g *for 18 h. The bands obtained (presumptive viruses) were pelleted by ultracentrifugation at 300,000 *g *for 2 h and pellets were dissolved in TN buffer for further tests.

### Viral nucleic acid extraction and amplification for shot-gun cloning

Total nucleic acid (DNA and RNA) was extracted from each band collected from the initial sucrose gradient separation using a High Pure viral nucleic acid kit (Roche, USA). For RNA extraction, samples were homogenized in Trizol^® ^reagent (Invitrogen, USA). RNA from the suspension was then purified using a Perfect RNA kit (Eppendorf, Germany). The first and second strand cDNA synthesis was carried out using the total nucleic acid extract as template and following the recommended protocol of a Marathon cDNA Amplification Kit (BD Biosciences, Clontech Inc. USA).

Target templates (containing cDNA plus DNA from the original extract) were amplified by touch-down PCR with an Advantage 2 Polymerase mix that included *Taq*Start Antibody for hot start PCR combined with Marathon kit primers that included an adaptor sequence for later cloning, as described in the system Advantage^®^2 PCR enzyme user manual (Clontech).

### Cloning and screening by blot hybridization

Direct cloning was achieved using either a TOPO T/A cloning kit (Invitrogen, USA) or a pGEM-T Easy (Promega, USA) cloning kit as directed by the manufacturer. Selected clones were screened by colony blots and dot-blot hybridization as previously described [1] using DIG-labelled total DNA extracted from healthy shrimp. Briefly, detection was performed with an alkaline phosphatase-conjugated anti-DIG antibody (Roche) at dilution of 1: 5000. Promising candidate clones (i.e., negative hybridization with the shrimp probe) were selected based on comparison to a negative control derived from plasmid DNA that produced a colorless dot.

### *In situ *hybridization

Tissues of specimens originating from MSGS ponds or experimental challenge tests and embedded in paraffin blocks were sectioned (5 μm) and subjected to *in situ *hybridization assays using candidate fluorescein-labelled probes selected for negative hybridization with shrimp DNA and lack of significant sequence identity with shrimp sequences or known pathogen sequences at GenBank. Random primed DNA labeling for LSNV was achieved following the protocol of a PCR fluorescein isothiocyanate (FITC) Fluorescein-High prime labelling kit (Roche, Germany) using a plasmid containing a 600 bp sequence of LSNV (Table 1). Candidate clones were labelled with a second chromophore rhodamine (tetrametyl rhodamine 5'dUTP)(Roche, Germany) in a similar manner. Specimens tested were those transferred from Davidson's fixative after 24-48 h of fixation to avoid degradation of RNA (Hasson et al 1997). *In situ *hybridization reactions were carried out as previously described (Sritunyalucksana et al., 2006). Briefly, the 2 oligo fluorescein labeled probes were mixed and applied to the specimens and incubated overnight at 42°C. The negative control comprised fluorescein without a labeled probe. After incubation, the specimens were washed and viewed with a confocal microscope.

### Sequence analysis

The clones were sequenced on both strands by Macrogen Inc. (South Korea). Fragments larger than 500 bp were sequenced from both ends, after which new primers were sequenced from the preliminary leads to fill gaps for both strands. Multiple alignments were performed using the programs DNA Baser (Heracle BioSoft S.R.L., Romania) and CLUSTAL W [21] to identify portions with 100% overlap so that contiguous sequence reads could be united into a single consensus sequence. Final sequences were subjected to BLAST 2.0 analysis of nucleic acid sequences and deduced amino acid sequences for 6 frames [22], using programs available online. These included nucleic acid and protein searches using BLAST (http://www.ncbi.nlm.nih.gov/BLAST/) and protein domain searches using InterPro 17.0 [23] and EMBL-EBI (http://www.ebi.ac.uk).

### DNase and RNase treatments

RNase A (Fermentas) and RNase-free DNase I (Fermentas) were used according to the manufacturer's instructions to determine whether the template nucleic acid for clones of interest was DNA or RNA. The nuclease digestions were performed in 10 μl volumes using 250 ng total nucleic acid from shrimp incubated for 30 min at 37°C. The nucleic acid from each treatment was extracted and purified using High Pure viral nucleic acid kits (Roche, USA) according to the manufacturer's direction. Nucleic acid templates were eluted from the columns using DEPC-treated water and used as for RT-PCR and PCR assays with the primers shown in Table 1.

### Northern blot analysis

RNA was extracted from the 21% CsCl gradient band and subjected to 0.8% agarose-formaldehyde gel electrophoresis before blotting onto a Biodyne nylon membrane (Pall Corporation). RNA extracted from SPF shrimp gills was also run in a parallel lane. The membrane was hybridized with DIG-labelled DNA probe prepared from ICE Probe 3. After incubation with anti-DIG antibody conjugated with alkaline phosphatase, hybridized signals were detected with NBT/BCIP substrate (Roche, Germany) according to the supplier's protocol.

### Shrimp bioassays

*Penaeus monodon *(5.132 ± 0.767 g, and 5.633 ± 0.798 g) obtained from the quarantine facility of the Shrimp Genetic Improvement Center at Chaiya in Surathani province were used for bioassays with inoculum derived from gradient centrifugation fractions of the tissue homogenates described above. These stocks had a history of freedom from HPV, MBV, yellow head virus (YHV) and WSSV for more than 6 generations. Using pleopod samples, they were tested again and found negative for the same viruses before challenge tests using standard PCR detection kits (i.e., IQ2000, GeneReach, Taiwan for YHV and WSSV and Easygene, Shrimp Biotechnology Business Unit, National Center for Genetic Engineering and Biotechnology, Thailand for HPV and MBV). Although tests for HPV and MBV are often carried out using DNA from hepatopancreatic tissues, our experience has shown that they can also be detected in DNA extracts of pleopod samples from infected shrimp. In addition, subsequent tissue sections prepared in parallel with those for our *in situ *hybridization reactions were stained with hematoxylin and eosin and showed no histopathology characteristic MBV or HPV in the hepatopancreas. The collected shrimp were acclimatized in the laboratory for 5 days in 65 L aquaria containing continuously aerated artificial seawater at 15 ppt and 30 to 35°C. There were 7 shrimp in each aquarium and 4 aquaria in each of the challenge and control groups (i.e., a total of 28 shrimp in each group). The control shrimp were each injected with 100 μl TN buffer only. The test shrimp were each injected with 100 μl of a 10^-6 ^dilution of a centrifuge fraction (21% CsCl w/w) that had been confirmed by one step RT-PCR to be positive for ICE using the primer set described in Table 1. The shrimp were subsequently reared for 60 days during which mortality, appearance and size were recorded. Shrimp collected at the moribund state were counted as dead. At 2 months post challenge, the gills of the injected shrimp were pooled and subjected to identical homogenization and gradient centrifugation as previously described. The 21% CsCl fraction was used for a repeated bioassay as described above. At the same time, the living shrimp from which gills had been removed were fixed as appropriate for analysis by light microscopy and TEM. Statistical analysis of shrimp weight, length and survival results were carried out using a simple Student t-test with SigmaStat 3.5 software (copyright Systat Software 2006). Differences were considered to be statistically significant when p ≤ 0.05.

## Authors' contributions

WP did most of the experimental and investigative work and assisted in preparation of the manuscript. SaS assisted WP with molecular methods and helped to design experiments and interpret the results. SiS did the electron microscopy and interpreted its results. BW helped with conceptualization of the work, experimental design and provision of shrimp for bioassays. TWF conceived the work, assisted in the experimental design and assumed the major role in interpretation of the results and preparation of the manuscript. All authors read and approved the final manuscript.

## Supplementary Material

Additional file 1 **Nortrhern blot analysis of RNA extract from the 21% CsCl gradient band**. Northern blot analysis of RNA extracted from the 21% CsCl gradient band (ICE) and from shrimp gills of specific pathogen free *P. monodon *(SPF) hybridized with DIG-labeled ICE Probe 3. M, Perfect RNA marker 0.2-10 kb (Novagen). The marker bands (length in nucleotides) are indicated to the left. Click here for file

Additional file 2 **Shrimp survival and growth from the 1st bioassay**. Details of survival, weight and length of test and control shrimp from the 1^st ^bioassay using inoculum from the 21% CsCl gradient band containing ICE. Click here for file

Additional file 3 ***In situ *hybridization negative control from bioassay #1**. Example of confocal photomicrographs of LO tissue from a buffer-injected control shrimp specimen from challenge test 1, negative for both LSNV and ICE by RT-PCR. Click here for file

Additional file 4 **Low magnification *in situ *hybridization of LO from bioassay #1**. Low magnification example of an *in situ *hybridization reaction using a rhodamine-labeled ICE probe (red) together with an FITC-labeled LSNV probe (green) with lymphoid organ (LO) tissue of a *P. monodon *specimen from challenge test 1 that was positive by RT-PCR for both ICE and LSNV. Click here for file

Additional file 5 ***In situ *hybridization negative control of eye from bioassay #2**. Example of confocal photomictographs of the fasciculated zone of the eye of a buffer-injected control shrimp specimen from challenge test 2, negative for both LSNV and ICE by RT-PCR. Click here for file
