# Supplementary material for: A novel integrase-containing element may interact with Laem-Singh virus (LSNV) to cause slow growth in giant tiger shrimp
Source: BMC Vet Res. 2011 May 14;7:18. doi: 10.1186/1746-6148-7-18 (PMC3117699; doi:10.1186/1746-6148-7-18)
Supplement: Additional file 1 — Nortrhern blot analysis of RNA extract from the 21% CsCl gradient band. Northern blot analysis of RNA extracted from the 21% CsCl gradient band (ICE) and from shrimp gills of specific pathogen free P. monodon (SPF) hybridized with DIG-labeled ICE Probe 3. M, Perfect RNA marker 0.2-10 kb (Novagen). The marker bands (length in nucleotides) are indicated to the left. [file 1746-6148-7-18-S1.DOC]

**Additional file 1 - Nortrhern blot analysis of RNA extract from the 21% CsCl gradient band**

Northern blot analysis of RNA extracted from the 21% CsCl gradient band (ICE) and from shrimp gills of specific pathogen free *P. monodon* (SPF) hybridized with DIG-labeled ICE Probe 3. M, Perfect RNA marker 0.2-10 kb (Novagen). The marker bands (length in nucleotides) are indicated to the left.

**
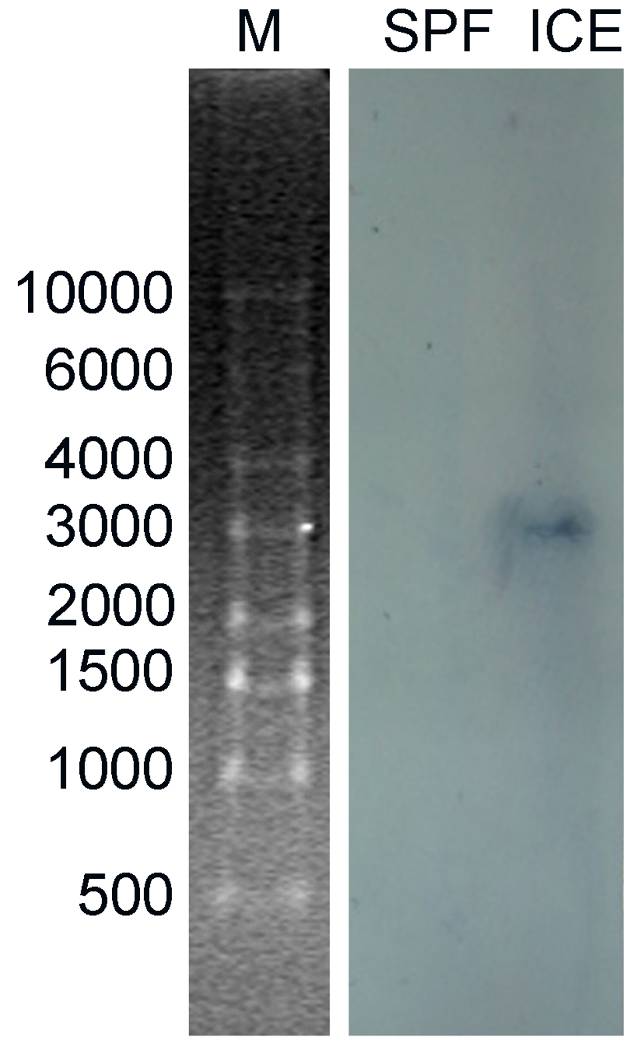
**
