# Supplementary material for: A novel integrase-containing element may interact with Laem-Singh virus (LSNV) to cause slow growth in giant tiger shrimp
Source: BMC Vet Res. 2011 May 14;7:18. doi: 10.1186/1746-6148-7-18 (PMC3117699; doi:10.1186/1746-6148-7-18)
Supplement: Additional file 2 — Shrimp survival and growth from the 1st bioassay. Details of survival, weight and length of test and control shrimp from the 1st bioassay using inoculum from the 21% CsCl gradient band containing ICE. [file 1746-6148-7-18-S2.DOC]

## Additional file 2. Shrimp survival and growth from the 1^st^ bioassay.

|  |  | **Control TN Buffer Injection** | | **Test CsCl band**  **(ICE) injection** | |
| --- | --- | --- | --- | --- | --- |
| **Replication** | **Parameters** | **1 Day** | **60 Days** | **1 Day** | **60 Days** |
|  | n | 7 | 5 | 7 | 4 |
| **1** | mean of body weight (g) | 4.7± 0.7 | 14.1 ± 2.1 | 5.1±0.6 | 9.0 ±2.8 |
|  | mean of length  (cm) | 4.6±0.5 | 10.3± 1.6 | 5.6±0.8 | 8.4±0.9 |
|  | n | 7 | 4 | 7 | 4 |
| **2** | mean of body weight (g) | 5.4±0.9 | 10.7± 1.6 | 5.1±1.0 | 10.0±1.5 |
|  | mean of length (cm) | 5.3±1.0 | 8.5±1.0 | 4.8±0.7 | 8.6±0.5 |
|  | N | 7 | 5 | 7 | 5 |
| **3** | mean of body weight (g) | 4.9±0.5 | 11.4±2.4 | 5.3±0.6 | 10.2±2.6 |
|  | mean of length (cm) | 5.3±0.6 | 8.6±0.4 | 5.3±0.6 | 8.9±1.0 |
|  | n | 7 | 4 | 7 | 4 |
| **4** | mean of body weight (g) | 5.3±1.0 | 13.6±3.1 | 5.1±0.7 | 11.8±1.6 |
|  | mean of length (cm) | 5.6±1.1 | 9.7±1.7 | 5.3±0.7 | 9.2±0.7 |
| Means of | mean of body weight (g) | 5.1± 0.3 | 12.5 ±1.4 | 5.3±0.3 | 10.3±1.0 |
| means ± SE | mean of length (cm) | 5.2± 0.4 | 9.3 ±0.8 | 3.7±0.1 | 8.8±0.3 |
| % mortality |  | 35.7 | | 38.6 | |
