# Supplementary material for: A novel integrase-containing element may interact with Laem-Singh virus (LSNV) to cause slow growth in giant tiger shrimp
Source: BMC Vet Res. 2011 May 14;7:18. doi: 10.1186/1746-6148-7-18 (PMC3117699; doi:10.1186/1746-6148-7-18)

**Additional file 3. *In situ* hybridization negative control from bioassay #1**

Example of confocal photomicrographs of LO tissue from a buffer-injected control shrimp specimen from challenge test 1, negative for both LSNV and ICE by RT-PCR. a) Phase image; b) Image of LSNV fluorescence probe showing two green spots of background noise; c) Image of ICE fluorescence probe showing the same two spots of noise; d) Combined images showing two co-localized, yellow spots of background noise.


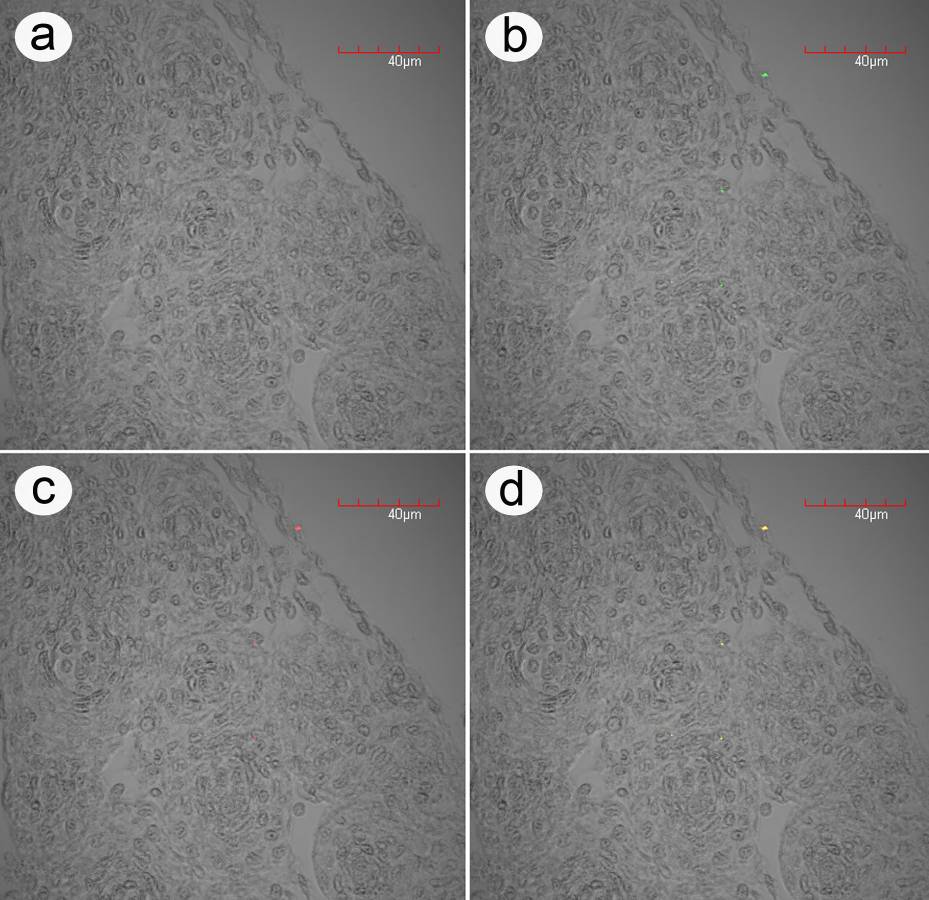

Supplement: Additional file 3 — In situ hybridization negative control from bioassay #1. Example of confocal photomicrographs of LO tissue from a buffer-injected control shrimp specimen from challenge test 1, negative for both LSNV and ICE by RT-PCR. [file 1746-6148-7-18-S3.DOC]
