# Supplementary material for: A novel integrase-containing element may interact with Laem-Singh virus (LSNV) to cause slow growth in giant tiger shrimp
Source: BMC Vet Res. 2011 May 14;7:18. doi: 10.1186/1746-6148-7-18 (PMC3117699; doi:10.1186/1746-6148-7-18)
Supplement: Additional file 4 — Low magnification in situ hybridization of LO from bioassay #1. Low magnification example of an in situ hybridization reaction using a rhodamine-labeled ICE probe (red) together with an FITC-labeled LSNV probe (green) with lymphoid organ (LO) tissue of a P. monodon specimen from challenge test 1 that was positive by RT-PCR for both ICE and LSNV. [file 1746-6148-7-18-S4.DOC]

**Additional file 4. Low magnification *in situ* hybridization of LO from bioassay #1**

Low magnification example of an *in situ* hybridization reaction using a rhodamine-labeled ICE probe (red) together with an FITC-labeled LSNV probe (green) with lymphoid organ (LO) tissue of a *P. monodon* specimen from challenge test 1 that was positive by RT-PCR for both ICE and LSNV. The images show the results of 5 layers combined. a) Phase image of tissue; b) Image of LSNV fluorescence; c) Image of ICE fluorescence; d) Combined images of a to c with a magnified insert showing that the fluorescence distribution for the two signals is single (green or red) and combined (yellow). Note that the fluorescence is located in the cell cytoplasm and is most intense in the normal tubules of the LO and much less intense in the spheroids (i.e., the circular groups of cells surrounded by a ring of sheath cells most clearly visible in the combined image d). Negative control images from a normal shrimp specimen are shown in Additional file 2.

**
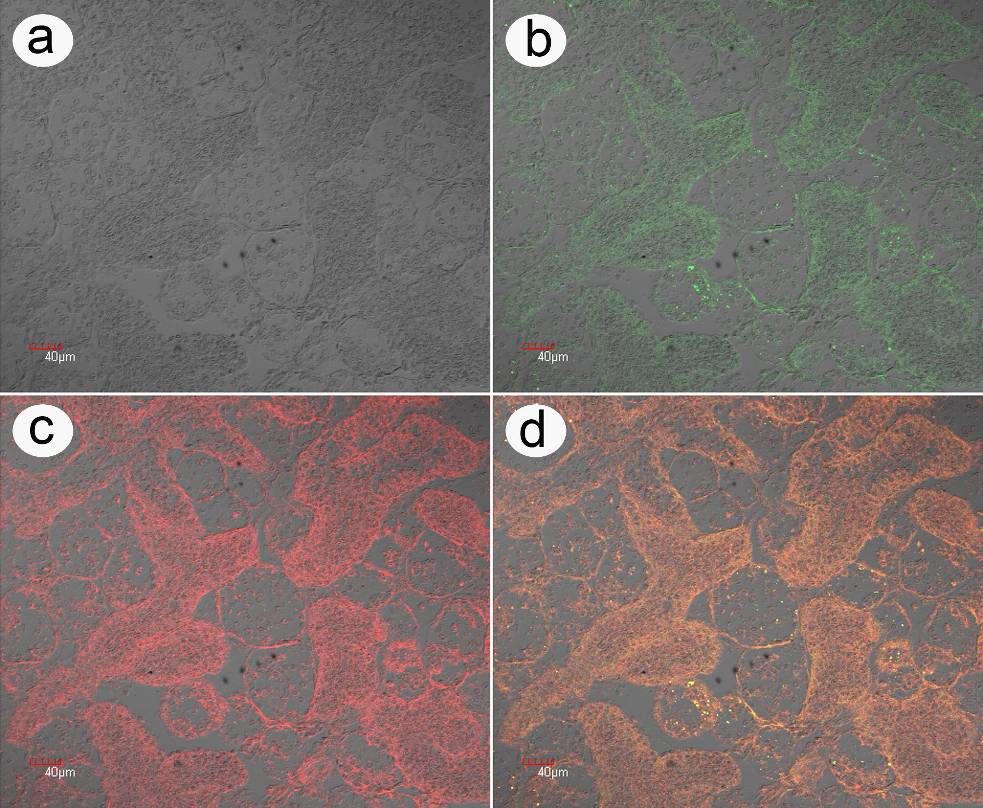
**
