# Supplementary material for: A novel integrase-containing element may interact with Laem-Singh virus (LSNV) to cause slow growth in giant tiger shrimp
Source: BMC Vet Res. 2011 May 14;7:18. doi: 10.1186/1746-6148-7-18 (PMC3117699; doi:10.1186/1746-6148-7-18)

## Additional file 5 - *In situ* hybridization negative control of eye from bioassay #2

Example of confocal photomictographs of the fasciculated zone of the eye of a buffer-injected control shrimp specimen from challenge test 2, negative for both LSNV and ICE by RT-PCR. a) Phase image; b) Image of LSNV fluorescence showing no signal in the fasciculated zone under the retinal layer that is below the false-positive fluorescence of the crystalline tracts and one piece of displaced crystalline tract in the fasciculated zone. c) Image of negative ICE fluorescence in the fasciculated zone with false-positive fluorescence as in image (b). d) Combined images showing co-localized (yellow) false-positive fluorescence for the crystalline tracts.


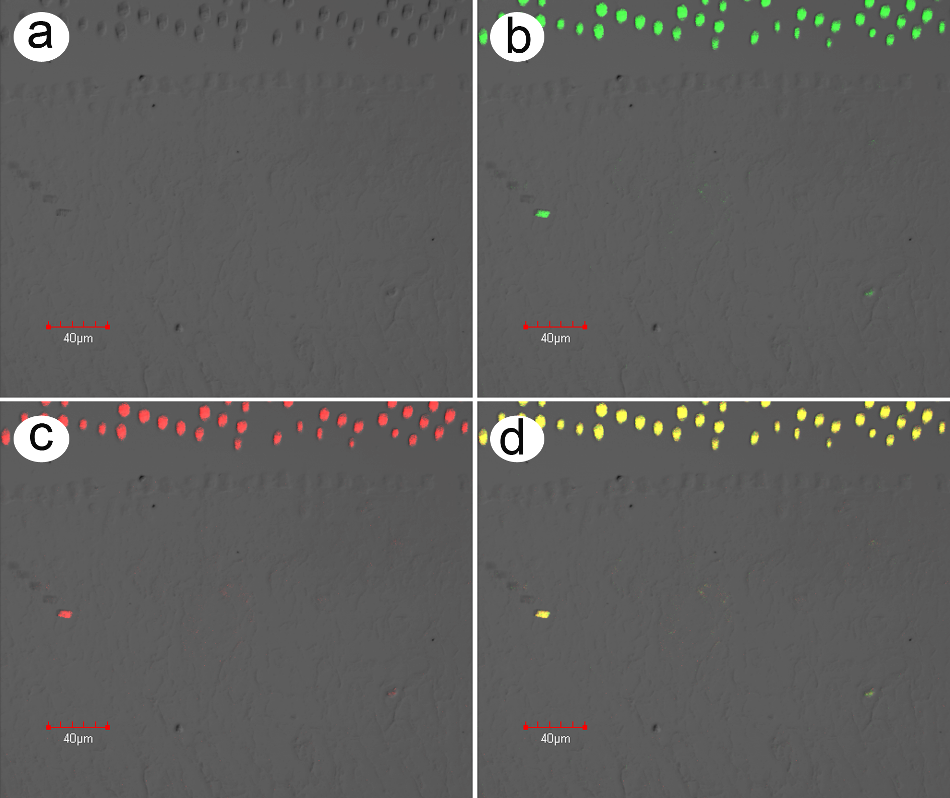

Supplement: Additional file 5 — In situ hybridization negative control of eye from bioassay #2. Example of confocal photomictographs of the fasciculated zone of the eye of a buffer-injected control shrimp specimen from challenge test 2, negative for both LSNV and ICE by RT-PCR. [file 1746-6148-7-18-S5.DOC]
